# The Effects of Antipsychotic Treatment on the Brain of Patients With First-Episode Schizophrenia: A Selective Review of Longitudinal MRI Studies

**DOI:** 10.3389/fpsyt.2021.593703

**Published:** 2021-06-24

**Authors:** Chengmin Yang, Jing Tang, Naici Liu, Li Yao, Mengyuan Xu, Hui Sun, Bo Tao, Qiyong Gong, Hengyi Cao, Wenjing Zhang, Su Lui

**Affiliations:** ^1^Huaxi MR Research Center, Department of Radiology, West China Hospital, Sichuan University, Chengdu, China; ^2^Department of Radiology, West China Hospital, Sichuan University, Chengdu, China; ^3^Functional and Molecular Imaging Key Laboratory of Sichuan Province, Psychoradiology Research Unit, Chinese Academy of Medical Sciences, West China Hospital, Sichuan University, Chengdu, China; ^4^Center for Psychiatric Neuroscience, Feinstein Institute for Medical Research, Manhasset, NY, United States; ^5^Division of Psychiatry Research, Zucker Hillside Hospital, Glen Oaks, NY, United States

**Keywords:** first-episode schizophrenia, antipsychotics, treatment response, longitudinal, magnetic resonance imaging

## Abstract

A large number of neuroimaging studies have detected brain abnormalities in first-episode schizophrenia both before and after treatment, but it remains unclear how these abnormalities reflect the effects of antipsychotic treatment on the brain. To summarize the findings in this regard and provide potential directions for future work, we reviewed longitudinal structural and functional imaging studies in patients with first-episode schizophrenia before and after antipsychotic treatment. A total of 36 neuroimaging studies was included, involving 21 structural imaging studies and 15 functional imaging studies. Both anatomical and functional brain changes in patients after treatment were consistently observed in the frontal and temporal lobes, basal ganglia, limbic system and several key components within the default mode network (DMN). Alterations in these regions were affected by factors such as antipsychotic type, course of treatment, and duration of untreated psychosis (DUP). Over all we showed that: (a) The striatum and DMN were core target regions of treatment in schizophrenia, and their changes were related to different antipsychotics; (b) The gray matter of frontal and temporal lobes tended to reduce after long-term treatment; and (c) Longer DUP was accompanied with faster hippocampal atrophy after initial treatment, which was also associated with poorer outcome. These findings are in accordance with previous notions but should be interpreted with caution. Future studies are needed to clarify the effects of different antipsychotics in multiple conditions and to identify imaging or other biomarkers that may predict antipsychotic treatment response. With such progress, it may help choose effective pharmacological interventional strategies for individuals experiencing recent-onset schizophrenia.

## Introduction

Schizophrenia is a chronic and severe psychotic disorder that affects approximately 1% of the population, and has a profound impact on individuals who have been affected (1). The core features of this disorder are positive symptoms (delusions and hallucinations), negative symptoms (apathy, loss of emotional expression) and cognitive impairments (2). Antipsychotics, which exert effects on eliminating these symptoms by interacting with the dopamine D2 receptors in the brain (3), have been the mainstream treatment for schizophrenia since the 1950s (4). However, in spite of development and administration for decades, the usage of antipsychotic drugs as the first choice for schizophrenia patients has been of sustain controversy. The reasons include: (1) low effectiveness for whole population-about 30% of first episode patients, as well as over 50% of patients with multiple episodes, did not respond to conventional antipsychotic treatment (5); (2) many side effects-specifically, first-generation (conventional) agents (FGAs) appear to have high efficacy in reducing positive symptoms but are accompanied with serious side effects, such as acute extrapyramidal side effects (EPS) and tardive dyskinesia (TD) (6), although these effects are shown to be attenuated by second-generation (atypical) agents (SGAs) (2); and (3) effects with unpredictable outcomes-it was found that patients exposed to antipsychotics appeared to have cell loss in brain and smaller brain volume (7–9), and what these changes might cause remains uncertain. Therefore, determining the effects of different antipsychotics on the brain is important to elucidate the efficacy and side effects of the drugs simultaneously, and to justify the administration of antipsychotics (10, 11).

The magnetic resonance imaging (MRI) provides an important alternative to examine how the antipsychotic treatment affects human brain *in vivo*. In previous work, using different imaging techniques and analytic algorithms, both structural and functional brain alterations were identified in response to antipsychotics in patients with schizophrenia, mainly involving the fronto-temporal brain regions (12), basal ganglia (13), limbic system, (14–16) and several key components within the default mode network (DMN) (17). However, the findings thus far have been inconsistent. While the discrepancy in previous findings might attribute to heterogeneity in sample size, patient ethnics, illness course, and analytic approaches, several other important factors were also showing great impacts on the brain changes, including different types of drug administered, duration of untreated illness, and follow-up time (the time period that the patients were treated). As a result, it is necessary to summarize findings between brain alterations and antipsychotic treatment, and to identify factors that might bias the effects. This is essential before validating the findings in larger samples with unified analytic methodology.

In such efforts, reviewing studies that have included patients with untreated schizophrenia and collected brain imaging data before and after treatment is preferable. However, given long-standing and almost universal treatment with antipsychotic medications right after illness onset especially in the Western countries, a great number of previous studies enrolled patients who had already taken antipsychotics for a period of time (8). From the research perspective, never-treated schizophrenia patients should be included at baseline to precisely identify the treatment effects of antipsychotics on the brain. Furthermore, patients with first-episode schizophrenia display a greater sensitivity, or respond easily, to antipsychotic treatment in reducing psychotic symptoms than those who with multiple episodes (5, 6, 18). Therefore, studies with antipsychotic-naïve patients with first-episode schizophrenia are especially important as a starting point for evaluating the brain alterations after the administration of antipsychotics, to better specify the anatomical and functional changes that are associated with treatment (19). With this in mind, over the past decade, our group has collected imaging and clinical data from a large sample of antipsychotic-naïve patients with first-episode schizophrenia and longitudinally followed these patients after treatment. We found that the gray matter volume in the hippocampus was reduced after 6 weeks of antipsychotic treatment in a dose-related manner (14), and the abnormal regional activities in the orbitofrontal cortex and occipital gyrus were partly normalized after 1-year treatment (20). These findings added importance evidence in this aspect, however, our patients were followed just for 1 year and findings from different centers varied a lot as noted above. Thus, the brain changes in response to antipsychotics remain uncertain and require further exploration and generalization.

When interpreting the treatment effects, it is noteworthy that the treatment effects could not be easily explained by the brain changes before and after treatment. The model associated with illness progression also needs to be taken into consideration since the relative contribution of both models (antipsychotic drug vs. illness progression) to different brain changes remains to be clarified. Neuroimaging studies have revealed progressive brain changes since first-episode schizophrenia and provided evidence that brain changes might represent an ongoing pathophysiological process (21, 22). Under this circumstance, taking the dynamics of brain structure and function within the illness itself into consideration is necessary in studies with longitudinal designs when brain changes along the treatment are assessed (22). Findings from these longitudinal designs should be interpreted with caution, as they are not necessarily representative of a causal effect of treatment but may involve progressive pathological changes along the illness duration, especially for patients who were followed for a long time.

In the current review, therefore, we attempted to identify and summarize longitudinal neuroimaging studies providing evidence for a relationship between antipsychotic medications and brain changes in patients with first-episode schizophrenia. For a better characterization, we focused our attention chiefly on studies with patients who had never received any antipsychotics (drug-naïve), rather than patients who had been free of effects of antipsychotic medication for a period of time before admission (drug-free).

## Materials and Methods

### Searching Procedures

This review was performed based on the Preferred Reporting Items for Systematic Reviews and Meta-Analyses (PRISMA) statement. We searched for publications in PubMed and Embase from Jan 1st, 2000 to the present. The key words used in the literature search were “first episode” or “first-episode” and “schizophrenia” and “antipsychotic treatment” or “antipsychotics” or “antipsychotic drug” or “antipsychotic medication” and “magnetic resonance imaging” or “MRI” or “diffusion tensor imaging” or “DTI” and “longitudinal.” We also limited the search to articles that were written in English, and manually checked the reference lists of the retrieved articles for additional relevant studies.

Studies were included based on the following criteria: (a) peer-reviewed original literature, (b) longitudinal MRI studies, (c) examining the effects of antipsychotic treatment on the brain, (d) drug-naïve or drug-naïve/drug-free patients, and (e) first-episode schizophrenia patients. Studies were excluded for any of the following reasons: (a) they were case reports, editorials, comments, reviews or meta-analyses, (b) MRI was not used as the main method to investigate the neural basis of schizophrenia, (c) the effects of antipsychotic treatment on brain changes of patients were not described, (d) patients had received antipsychotic medication at the baseline assessment, or (e) patients studied had disorders other than schizophrenia, such as bipolar disorder, depression, brief psychotic disorder, delusional disorder or psychotic disorder not otherwise specified.

**Figure 1:**
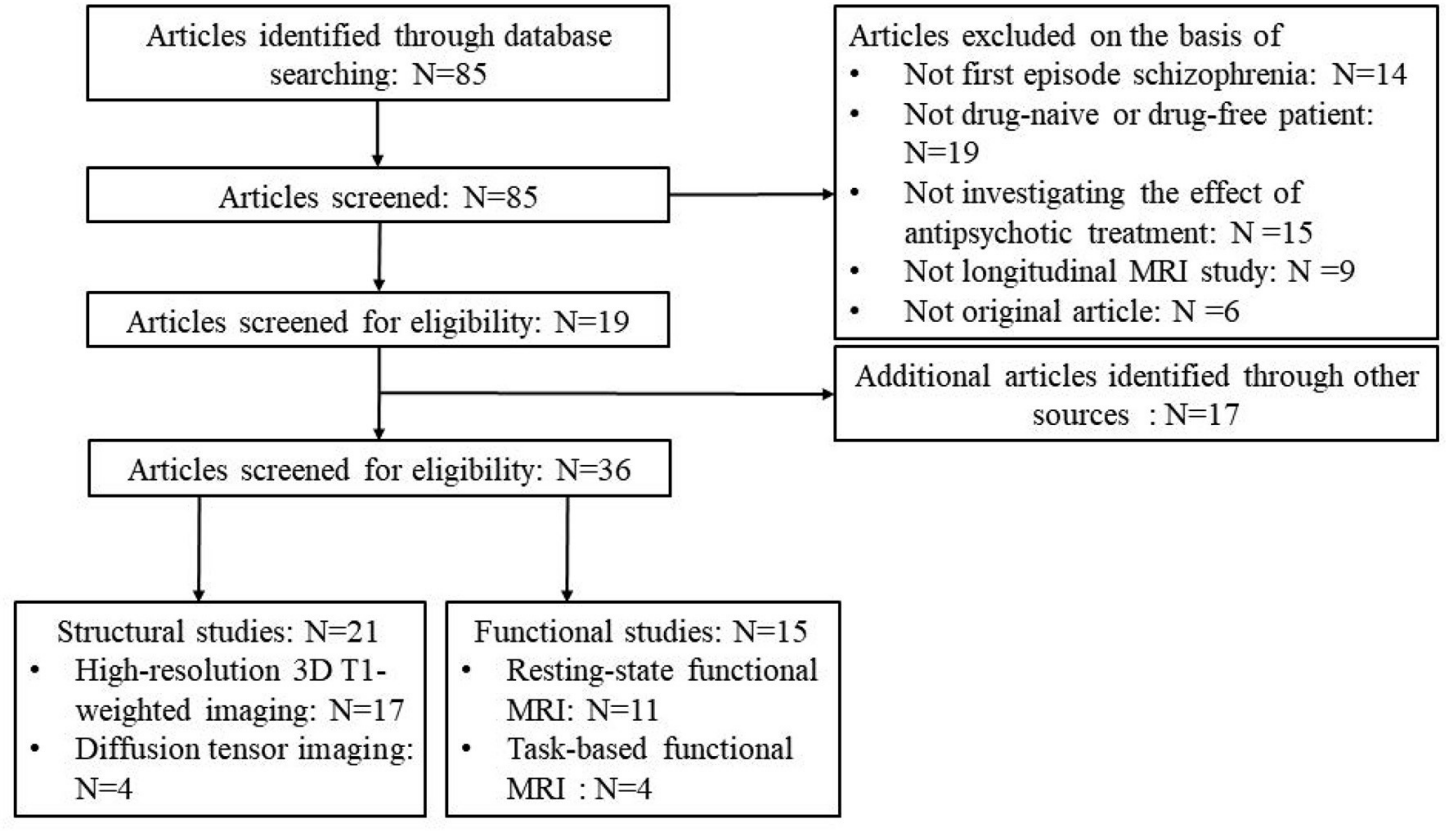
Flowchart of the article search and selection procedure for this review.

Notably, to better specify the brain changes associated with anatomy and function, respectively, we stratified the included studies into those were of structural imaging study and functional imaging study (Figure 1). In structural imaging studies, high-resolution 3D T1-weighted imaging and diffusion tensor imaging were collected, and structural parameters for gray matter (gray matter volume, cortical thickness, surface area, and etc.) and white matter [fractional anisotropy (FA), mean diffusion (MD) and etc.] were assessed. In functional imaging studies, resting-state or task-based functional MRI data was acquired, and imaging measures of regional activity, functional connectivity between regions, and brain networks were calculated.

### Quality Assessment of Selected Studies

The quality of each included study was evaluated with Newcastle Ottawa Scale (23), which contains 3 categories: 4 items for patients selection, 1 item for study comparability and 3 items for outcomes assessment. The score is classified into 3 scales: 7–9 defined as good, 5–6 is fair quality, and 0–4 is poor quality. Of note, this checklist was just used to assess the quality of the included studies in our review, without criticizing the work itself or investigators.

## Results

A flowchart of the selection procedure with strict inclusion and exclusion criteria is shown in Figure 1. Among the 85 studies that were found according to our searching strategy, 19 (14, 15, 20, 24–39) of them met the inclusion criteria and were enrolled. Additional 17 (16, 17, 40–54) studies were found in other sources: five studies (50–54) came from a review (55), 4 studies (16, 46–48) came from another review (19), the co-author of 3 studies (40, 43, 45), 2 studies (17, 41) and one study (42) came from three research centers, respectively, which were same as the included studies through our searching strategy, one study (44) came from our group and one study (49) came from a reference list of a included study (37). All additional studies included 9 structural studies (16, 44, 48–54) and 8 functional studies (17, 40–43, 45–47). Finally, a total of 36 longitudinal studies was included in this review with good quality (mean score = 8.78) (Supplementary Table 1), including 21 studies (14–16, 27–30, 32, 33, 35–37, 39, 44, 48–54) on brain structure and 15 studies (17, 20, 24–26, 31, 34, 38, 40–43, 45–47) on brain function (Figure 1).

In 10 studies patients were treated with both FGAs and SGAs after baseline MRI scan (14, 15, 20, 28, 37, 39, 47–49, 52). Other 24 studies observed brain changes after treatment with SGAs (16, 17, 24–27, 30–36, 38, 40, 41, 43–46, 50, 51, 53, 54). Only two studies had no clear information of medication (29, 42). Most first-episode schizophrenia patients were in their 20 s (mean age) with illness duration <3 years, except one study with over 3 years (48). At the baseline scan, 34 studies recruited patients who had been drug-naïve (14–17, 20, 24–33, 35–44, 46–54), and the other two studies had mixed sample with both drug-naïve and drug-free patients (34, 45). The periods of follow-up ranged from 1 week to 4 years. To facilitate the discussion, the time period of antipsychotic treatment was divided into 3 phases as indicated in a previous work (56). In particular, the short-term phase was defined as the period of acute and intense psychosis, and typically <1 year. The mid-term phase was defined as the 2- to 3-year period after the acute phase, and the long-term phase was defined as the period from 3 years onward (56).

### Structural Imaging Studies

Among structural MRI studies, 17 studies investigated gray matter volume alterations (14–16, 27–30, 35, 36, 39, 48–54). In particular, four studies used Diffusion Tensor Imaging (DTI) to assess the white matter microstructure (32, 33, 37, 44). Structural changes in the brain of patients with first-episode schizophrenia before and after treatment have been frequently reported in the frontal and temporal gyrus, basal ganglia and limbic system. These brain alterations were associated with symptom improvements, also influenced by antipsychotic dose. However, the effects of antipsychotics on the brain anatomy were inconsistent. Specifically, the gray matter volume in the frontal and temporal regions decreased after mid-term and long-term treatment (39, 48) but increased after short-term following-up (30, 49, 50). One of these studies observed that greater volume of the prefrontal cortex was associated with improvement in negative symptoms (30). Regarding the striatum, amisulpride monotherapy induced hypertrophy of the structure (27) but quetiapine monotherapy caused hypotrophy (16). In these two studies, it was showed that the reductions in positive symptoms were associated with striatal volume increases after the administration of antipsychotics (27), while striatal volume loss was related to low quetiapine dose (16). As to the subregions of striatum, such as the caudate and putamen, their gray matter consistently increased in patients after antipsychotic treatment (15, 49, 53, 54). Interestingly, there was a significant sex effect on the relationship between atypical neuroleptic exposure and changes in the structure of the caudate over time (54). In women, greater amount of drug exposure was associated with less enlargement of the caudate, with the opposite observed in men. The volume of the anterior cingulate cortex (ACC) was decreased in patients treated with mixed FGAs and SGAs (28) or SGAs (52) but increased with FGAs (52). The increased ACC volume was correlated with greater psychotic symptom improvements (52). In addition, most studies showed reduced gray matter volume in the hippocampus over time (14, 16, 29, 35), and the changes altered in a dose-related manner (14, 16). One study observed progressive gray matter increase in the amygdala-hippocampal cluster that was related to drug plasma levels (36). Despite the limited number of DTI studies, two studies (37, 44) reported widespread white matter integrity deficits in patients after treatment, while another study revealed that lower fractional anisotropy (FA) was normalized in patients treated with amisulpride monotherapy (33). A graph-based study for anatomical brain networks showed deficits of topological parameters in the limbic system were normalized along with reductions in positive symptoms after 8 weeks of risperidone monotherapy (32).

### Functional Imaging Studies

Among functional MRI studies, six of them measured regional activities of brain regions (24, 31, 34, 38, 43, 46), including those at resting-state and task-based conditions. Seven studies calculated functional connectivity between regions within the brain (17, 25, 26, 40–42, 45), and two studies involved both functional measures mentioned above (20, 47). Antipsychotics could manifest acute effects on brain function after a very short period of antipsychotic treatment, as reported in studies where patients were treated for only 1 week (40, 43). However, the findings are diverse, different patterns of functional changes were showed that might relate to different mechanisms or pathological processes. Several studies have reported that brain abnormalities at baseline, mostly involving the frontal, parietal, and temporal cortices, basal ganglia and limbic system, were normalized or reduced after being treated (17, 20, 24–26, 34, 38, 40, 41, 43, 47). These dynamic changes in abnormal brain activation with symptom improvements were observed after treatment, which may indirectly represent the beneficial effects of antipsychotics. However, the normalization of dysfunction observed before treatment did not occur in all brain regions or networks in first-episode patients after treatment. The dysfunction might be stable in brain regions over time, mainly including the caudate, putamen and dorsolateral prefrontal cortex (DLPFC) (31, 34, 42, 46). Additionally, the DLPFC was unimpaired prior to treatment but showed significantly reduced activation after treatment, suggesting that antipsychotics may have adverse effects on brain function (34).

## Discussion

The present review article summarized the effects of antipsychotics on brain structure and function in patients with first-episode schizophrenia. The longitudinal brain changes in relation to antipsychotic treatment remain inconclusive, as inconsistency was shown among results from different studies which may be attributable to differences in the study design. Moreover, the common brain regions were frequently reported. Specifically, basal ganglia function, especially striatum, as fundamental of dysregulated dopaminergic modulation is underlying the symptoms of schizophrenia, which is affected by antipsychotic treatments (57). Disrupted DMN plays an important role in the pathogenesis of schizophrenia, and the network are plastic as well as can respond to effective medications (58). Here, we discuss three main factors and their potential effects on the results, namely, the type of drugs, the follow-up period and the duration of untreated psychosis (DUP).

### Effects of Different Types of Antipsychotics

The different profiles of affinity for dopamine D2 receptors of FGAs and SGAs may result in different brain changes. FGAs have affinity for dopamine D2 receptors, which are highly expressed in the basal ganglia (59). However, SGAs have a favorable ratio of serotonin 5-HT2A to D2 antagonism, therefore, the findings of treatment effects on the brain have been inconsistent. Specifically, in a functional MRI study (31), patients with risperidone monotherapy showed increased synchronous neural activity in the putamen, while greater increase in activation in the putamen related to less improvement in positive symptoms. In contrast, another study (43) with olanzapine monotherapy reported decreased synchronous neural activity in the putamen, and the activation reduction in the putamen was positively related to improvements in positive symptoms. Besides putamen, the volumes of caudate nuclei and accumbens were found to be increased in patients with risperidone monotherapy, but striatal reductions were observed in patients with quetiapine monotherapy (16, 53). The putamen, caudate nuclei and accumbens are parts of the striatum with rich dopamine receptors, which are recognized as antipsychotic treatment targets for schizophrenia (15). Regarding pharmacological mechanisms, risperidone has high affinity for both 5-HT2A and dopamine D2 receptors (60). Meanwhile, olanzapine has high affinity for the 5-HT2A receptor and moderate affinity for the D2 receptor (61). The differences in therapeutic targets between olanzapine and risperidone may cause opposite findings in the above studies.

Inconsistent findings were also shown in first-episode schizophrenia after SGAs at the network level (17, 45). Specifically, the functional connectivity between the posterior cingulate cortex (PCC) and precuneus (PCUN) was found either increased (45), or decreased (17) in patients before treatment in different studies, but such changes were found to be normalized or reversed in different directionality after treatment in tandem with the improvement in symptoms, i.e., the pre-treatment increased/decreased functional connectivity was decreased/increased after treatment (17, 45). Both PCC and PCUN are central nodes for the default-mode system (62), and are considered to be closely related to functions of self-awareness, self-centered mental imagery, and extraction of episodic memory (63). The DMN was a network circuit, in which the abnormalities were commonly thought to be associated with the core model of the psychopathology of schizophrenia (64). The antipsychotics appear to exert the therapeutic effects greatly in fixing the changes in DMN, which could happen universally after initiation of antipsychotics in schizophrenia patients.

Notably, different drugs might be administered simultaneously in clinical practice for those who with first-episode schizophrenia. In this situation, the structural and functional brain changes observed might represent combining effects of differential antipsychotics. The explanation of effects from different drugs could be difficult. In our review, most patients in the included studies were treated with multiple antipsychotics. Among these studies, consistent findings of reduced volumes in the hippocampus (14, 16, 29) and ACC (28, 52) were observed. In particular, increased FGA exposure was correlated with increased ACC volume, and increased SGA exposure was related to decreased ACC volume over time (52). Other studies did not find the same pattern, but they only focused on regions within dopaminergic projections. While changes or associations could be observed in these studies, the complicated interactions between drugs or also with other factors make it hard to determine where the observed effects come from.

### Effects of Follow-Up Periods

The follow-up periods in the reviewed studies ranged from 1 week to 4 years, including 31 short-term studies, four mid-term studies and only one long-term study according to the abovementioned definition. Short-term follow-up studies with different antipsychotic treatments reported robust brain structural and functional changes in first-episode schizophrenia, mainly involving the fronto-temporal cortex, basal ganglia, limbic system and several key components in the DMN. There are relatively fewer mid-term and long-term follow-up studies, and these studies revealed gray matter loss in the frontal and temporal gyri (39, 48) and whole brain volume (8, 65). A longer follow-up or treatment exposure was associated with a greater decrease in brain volumes, and greater intensity of antipsychotic treatment was associated with brain volume reduction (65).

In a recent functional brain network study (66), it was investigated that whether functional alterations in the brain were different after long-term treatment in schizophrenia patients with or without prior antipsychotic treatment. Long-term treatment may protect the functions of the amygdala, hippocampus, and striatum. Although this cross-sectional study should be interpreted with caution, it provided some new insights into the clinical benefit of long-term treatment, particularly in disease-related regions. In addition, compared to the structural changes associated with hippocampal deficits in patients receiving short-term treatment, a study identified advantageous effects of long-term antipsychotic treatment in hippocampal subfields (67). Furthermore, white matter microstructure abnormalities in the corpus callosum were investigated in patients with first-episode schizophrenia after acute antipsychotic treatment (44), while one of our previous studies showed that long-term antipsychotic treatment may also exert positive effects on the integrity of the corpus callosum (68). It has also been postulated that longer antipsychotic treatment may be associated with more adverse effects, such as weight gain, metabolic disturbance or insufficient adherence (69). However, the effects observed above come from a cross-sectional study, and longitudinal studies are needed to confirm this phenomenon and elucidate the causes.

### Effects of the DUP

DUP is defined as the time from emergence of psychotic symptoms until treatment with antipsychotics, which usually ranges from 8 to 48 weeks. The DUP has been shown to be positively associated with worse treatment responses (70). Therefore, patients with first-episode schizophrenia are recommended to take appropriate antipsychotics as soon as possible (71). In a structural study (72), smaller hippocampal gray matter volume was associated with worse cognitive performance, which might be mediated though the dopamine pathway (73). Specifically, longer DUP was significantly associated with smaller whole hippocampal volume (74). Another study (75) observed a positive association between longer DUP and accelerated hippocampal atrophy during initial treatment. These findings together may suggest that impairments in brain structures are associated with both poorer outcomes in schizophrenia and longer DUP. Therefore, reducing the DUP and providing early pharmacological intervention is critical to preventing structural deficits and improving clinical outcomes (74).

In this review, most studies employed young adults with DUP of <1 year. One functional study suggested that longer DUP led to more severe disruptions in functional connectivity in the sensory-motor network, which may subsequently result in difficulties in the treatment of positive symptoms (42). However, none of the included functional studies analyzed patients with long DUP (>2 years). One cross-sectional study (76) compared two cohorts with different DUPs (2 weeks to 2 years) and found that longer DUP was correlated with worse treatment responses and overall decreased functional connectivity between striatal nodes and specific regions within frontal and parietal cortices. Based on these results, the antipsychotics exerted different effects that might be related to different DUPs on patients as revealed by functional changes in the brain. However, the interpretation of these results should consider the heterogeneity of patients between cohorts.

## Potential Limitations

There are some limitations that need to be noted. First, longitudinal studies investigating the long-term effects of antipsychotic treatment on brain are difficult, and therefore only few longitudinal imaging studies have been conducted to date. Second, the studies included in this review were published with a range of 20 years, therefore different imaging techniques and analyses were used which may influence the results. Third, our review only focused on treatment effects related to type of drugs, follow-up periods, and the DUP. Other variables or factors that may potentially relate to the treatment effect were not discussed due to limited number of studies, such as a history of substance abuse. Fourth, The site effects may to certain degree account for the variability between studies.

## Future Directions

While conducting longitudinal studies is essential to evaluating medication effects, general effects of antipsychotic medication should be performed in studies controlling factors such as antipsychotic type, DUP, and follow-up period, and the generalization of the findings should be tested in independent datasets. In addition, specific effects associated with different follow-up periods, variable DUP and other factors should also be examined, preferably at the individual level in order to promote individualized medications.

## Conclusion

To sum up, several brain regions in patients with first-episode schizophrenia are consistently reported and related to the effects of antipsychotics, including the frontal and temporal lobes, basal ganglia, limbic system, and some key components within the DMN. However, the specific anatomical and functional changes in these regions differ between different types of antipsychotics, follow-up periods and DUPs. Future studies with larger and more homogeneous patient samples are needed to clarify the effects of different antipsychotics in various conditions, which might help to develop pharmacological and interventional strategies for individuals experiencing recent-onset schizophrenia.

## Author Contributions

SL and WZ contributed to the conception and design of the study, as well as the supervision of all the work of this review. CY, JT, and NL contributed to literature searching and drafting of the manuscript. All authors made critical revision of the manuscript for important intellectual content and gave final approval of the version to be submitted.

## Conflict of Interest

WZ consulted to VeraSci. The remaining authors declare that the research was conducted in the absence of any commercial or financial relationships that could be construed as a potential conflict of interest.
