# Supplementary material for: The Effects of Antipsychotic Treatment on the Brain of Patients With First-Episode Schizophrenia: A Selective Review of Longitudinal MRI Studies
Source: Front Psychiatry. 2021 Jun 24;12:593703. doi: 10.3389/fpsyt.2021.593703 (PMC8264251; doi:10.3389/fpsyt.2021.593703)
Supplement: Supplementary file 1 [file Table_1.DOCX]

Supplementary Material

**Table S1**. Main findings of the antipsychotics effects of longitudinal structural and functional studies in first episode schizophrenia as well as the quality assessment of all included studies.

| Author/Publication year | Subjects Number (Male)  Age mean, SD | Illness duration (months) | medication state at baseline | Diagnostic criteria | Following time | Type of medicine | Main findings | Quality assessment (NOS) |
| --- | --- | --- | --- | --- | --- | --- | --- | --- |
| Li, et al. (2018)(1) | 41 (17) FES, 23.90±7.72 39 (19) HC, 24.01±8.18 | 8.99 ±12.65 | naïve | DSM-IV-TR | 6-week | FGA/SGA:  Risperidone  Olanzapine  Clozapine  Quetiapine  Aripiprazolea  Sulpiride | 1. baseline: ↑GMV  1) bilateral molecular layers of the hippocampus  2) bilateral granular cell layers of the dentate gyrus  3) bilateral cornu ammonis area 4 2. follow-up: ↓GMV  1) bilateral total hippocampus with subfield volume reduction noted in previously enlarged subfields  2) bilateral hippocampal tails  3. Subfields with volume increases before treatment were reduced to the level of healthy controls or near to it after treatment. 4. These abnormalities were reduced after acute antipsychotic therapy in a dose-related manner together with volume reductions in other areas that were not hypertrophic before treatment. | 9 |
| Rizos, et al. (2014)(2) | 14 (8) FES, 29.71 ± 10.21  0 HC | 8.00 ± 4.60 | naïve | DSM-IV | 8 months | SGA:  Olanzapine  Quetiapine  Risperidone  Aripiprazole  Amisulpride | 1. follow-up: ↓GMV: left hippocampal 2. The higher the BDNF levels change the higher were the differences of corrected left hippocampus after 8 months of treatment with atypical antipsychotics. | 9 |
| Ebdrup, et al. (2011)(3) | 22 (15) FES, 26.2±5.4 28 (21) HC, 28.4 ±6.0 | 44.5 ± 54.75 | naïve | DSM-IV | 6-month | SGA: Quetiapine | 1. follow-up: ↓GMV: bilateral striatal and hippocampal 2. The higher baseline positive symptoms were associated with more striatal and hippocampal loss over time. 3. Striatal volume loss was most pronounced with low quetiapine doses and less apparent with high doses. 4. Hippocampal volume loss appeared more pronounced with high quetiapine doses than with low doses. | 9 |
| Lei, et al. (2019)(4) | 34 FES  (20 (12) NDS;14(10) DS), 22.20 ± 6.65; 21.79 ± 5.35 32 (23) HC, 21.59 ± 4.65 | <1 year | naïve | DSM-IV | 1-year | Antipsychotic (NA) | 1. baseline: DS group:  1) ↓WMV: bilateral posterior limb of the internal capsule (PLIC) and cerebellar tonsil  2) ↓GMV: cerebellar culmen  2. follow-up:   1) NDS group:   ↑GMV: left thalamus; ↓GMV: frontal and cingulate cortex  ↓WMV: bilateral PLIC  2) DS group: showed no progressive WMV changes  3) both groups: ↓GMV: hippocampus and insular cortex 3. Double dissociations in developmental brain volume changes in the first year after clinical contact for psychosis in DS versus NDS patients. | 8 |
| Poeppl, et al. (2014)(5) | 20 (15) FES, 27.75±7.10 30 (19) HC, 30.20±7.61 | NA | naïve | DSM-IV-TR  ICD-10 | 3-week | SGA: Quetiapine | 1. Quetiapine plasma level correlated positively with GM increase in the amygdalohippocampal cluster, in particular the laterobasal amygdalar subdivision. 2. Small volume correction (SVC) indicated that quetiapine level-related GM increase in the amygdala was significant on voxel level | 8 |
| Andersen, et.al. (2020)(6) | 21 (10) FES,  23.5 ± 4.8 23 (12) HC, 24.1 ± 5.01 | 20.2±48.1 | naïve | ICD-10 | 6-week | SGA: Amisulpride | 1. Striatal increase was predicted by amisulpride dose. 2. A significant reduction in symptom severity was observed at a mean dose of 233.3 (SD = 109.9) mg, corresponding to D2/3R occupancy of 44.65%.  3. Reduction in positive symptoms correlated significantly with striatal volume increase | 9 |
| Yue, et al. (2016)(7) | 20 (10) FES, 24.45 ±5.51 24 (13) HC, 24.79 ±6.11 | 22.92±23.28 | naïve | ICD-10 | 8 weeks | SGA:  Clozapine Quetiapine Aripiprazole Olanzapine | 1. baseline: ↓GMV: right superior temporal gyrus 2. follow-up: ↑GMV: bilateral prefrontal cortex, insula, right thalamus, left superior occipital cortex, bilateral cerebellum 3. Greater enlargement of the prefrontal cortex is associated with the improvement in negative symptoms.  4. A more enlarged thalamus is associated with greater improvement in positive symptoms. | 9 |
| Molina, et al. (2005)(8) | 17 FES, 25.6 ± 4.0 11 HC, 28.4 ± 6.2 | 27.6 ± 16.8 | naïve | DSM-IV paranoid schizophrenia | 2-year | SGA: Risperidone | 1. GM increases and WM decreases in the parietal and occipital lobes 2. The greater the initial deficit, the greater the increase in GM 3. The greater the initial excess, the greater the longitudinal decrease WM | 9 |
| Deng, et al. (2009)(9) | 20 (9) FES， 29.9 ±13.5 (within 3 weeks)  26 ±10.0 (beyond 3 weeks) 11 (5) HC, 28.0 ±11.7 | NA | naïve | DSM-IV | 3 weeks (10) 8 weeks (10) | FGA/SGA:  Amisulpride Olanzapine Risperidone Quetiapine Trifluoperazine Flupenthixol Haloperidol | 1.After 3 weeks of anti-psychotic treatment, significant grey matter volume increased in the right caudate, superior and inferior frontal gyrus, precentral gyrus, and left inferior parietal lobule . 2.After 8 weeks of anti-psychotic treatment, volume increased in the right thalamus and bilateral cerebellum . 3.Significant grey matter reduction was detected in the left medial frontal gyrus at both 3- and 8-week intervals. 4.We speculate that drug-mediated neuroplasticity may provide a biomarker for clinical recovery. | 8 |
| Heitmiller, et al. (2004)(10) | 14 (7) FES, 26.3 ± 6.8 14 (7) HC, 26.7±11.3 | 46 ± 50.34 | naïve | DSM-IV | 30.2 ±13.3 months  (>2 years) | SGA:  Risperidone  Olanzapine  Quetiapine  Clozapine | 1. There was no difference between patients and controls in the amount of change over time in the volume of the caudate.  2. A significant gender effect on the relationship between atypical neuroleptic exposure and changes in the structure of the caudate over time. 3. For woman, the greater the amount of drug exposure, the less enlargement; For man, the greater the drug exposure, the greater the enlargement in volume | 9 |
| Massana et al. (2005)(11) | 11 (8) FES, 23 ± 4 15 (7) HC, 23.7 ± 3.1 | NA | naïve | DSM-IV | 3 months | SGA: Risperidone | 1.↑GMV: right and left caudate nuclei and the left accumbens 2. Treatment with risperidone was associated with an increase in basal ganglia volume | 8 |
| Li, et al. (2012)(12) | 40 (17) FES, 24.05±7.75 23 (14) HC, 22.49±5.83 | 9.33 ± 12.72 | naïve | DSM-IV | 6-week | FGA/SGA:  Risperidone  Olanzapine  Quetiapine Aripiprazole  Sulpiride  Haloperidol | 1. follow-up: ↑GMV: (FES vs. HC): right putamen 2. The volume change in the right putamen was found to be positively correlated with the reduction ratio of positive symptoms | 9 |
| Li, et al. (2019)(13) | 63 (24) FES, 24.17 ±8.14 0 HC | 7.96 ± 11.46 | naïve | DSM-IV | 1-year | FGA/SGA:  Risperidone  Olanzapine  Quetiapine Aripiprazole  Sulpiride  Haloperidol | 1. ↓GMV: anterior cingulate cortex (ACC), insula and inferior frontal gyrus (IFG), superior temporal gyrus (STG), middle temporal gyrus (MTG), precuenus, and dorsolateral prefrontal cortex (DLPFC) reduced with time in patients. 2. The lower volume of insula and IFG at baseline predicted a lack of improvement in positive and disorganization symptoms. | 9 |
| McCormick, et al. (2005)(14) | 22 (16) FES on atypicals 29.3 ± 5.02 9 (7) FES on typicals 27.1 ± 7.71 11 (5) HC 30.5 ± 6.93 | 27.5 ± 8.7 31.7 ± 18.2 | naïve | DSM-IV | 2-3 years | FGA/SGA:  Risperidone Olanzapine Clozapine Haloperidol Perphenazine Fluphenazine Thiothixene | 1.Increased typical neuroleptics exposure over time was correlated to increased anterior cingulate volume over time 2.Increased atypical neuroleptics exposure was correlated to decreased anterior cingulate volume 3.Increased anterior cingulate volume was correlated to greater psychotic symptom improvement | 9 |
| Theberge et al. (2007)(15) | 16 (14) FES, 25 ± 8 16 (14) HC, 29 ± 12 | 24 ± 27 | naïve | DSM-IV schizophrenia or  schizophreniform psychosis | 10 months  / 30 months | FGA/SGA:  Olanzapine Risperidone Quetiapine Haloperidol Ziprasidone Clozapine | 1.↓ GMV in left precuneus at 10 months 2. ↓ GMV in left precuneus (expanded to frontal, temporal, parietal and limbic lobes) at 30 months 3. Parietal and temporal lobe grey matter loss was correlated with thalamic glutamine loss. | 9 |
| Mané et al. (2009)(16) | 15 (12) FES, 25.56 ±5.77 11 (8) HC, 30.31 ± 4.36 | 5.78 ± 5.55 | naïve | DSM-IV  schizophreniform  disorder or  schizophrenia | 4 years | FGA/SGA:  Risperidone Olanzapine Quetiapine  but one of them was treated with Zuclopenthixol. | 1.(difference maps versus HCs’ difference maps) ↓ GMV in FES: left superior temporal gyrus, right orbitofrontal gyrus; ↑ GMV in FES: bilateral lingual gyrus and right cuneus. 2. Gray matter changes in patients in the left lingual gyrus, right insula and right cerebellum, were inversely related to functional outcome. | 9 |
| Girgis et al. (2006)(17) | 15 (7) FES, 23.6 ± 5.9 15 (7) HC, 23.7 ± 3.1 | 26.3 ± 23.7 | naïve | DSM-IV  schizophrenia, schizoaffective and schizophreniform disorders | 6-week | SGA: Risperidone | 1.↑GMV: left superior temporal gyrus (STG) and middle temporal gyrus (MTG); ↓GMV: left rectal gyrus; ↓WMV: corpus callosum (CC) 2. Risperidone has short-term effects on brain parenchyma in individuals with first-episode psychosis. | 9 |
| Meng, et al. (2019)(18) | 35 (16) FES, 23.83 ± 6.96  19 (12) HC, 21.05 ± 5.10 | 7.47 ± 9.88 | naïve | DSM-IV | 6-week | SGA:  Risperdal  Aripiprazole  Olanzapine  Clozapine  Quetiapine | 1. follow-up: ↓FA: bilateral posterior corona radiata, bilateral anterior corona radiata, bilateral superior corona radiata, bilateral posterior thalamic radiation (including the optic radiation), left posterior limb of the internal capsule, right posterior corona radiata emanating from precuneus and body of the corpus callosum. 2. Dosage of antipsychotic medications was positively correlated with FA changes in two clusters of the right superior corona radiata. | 9 |
| Ebdrup, et al. (2016)(19) | 38 (28) FES, 25.9 ± 6.5 38 (26) HC, 25.8 ± 6.4 | 18.75 ± 18.13 | naïve | ICD-10/SCAN schizophrenia or schizoaffective psychosis | 6 weeks | SGA: Amisulpride | 1. baseline: ↓FA: right anterior thalamic radiation (ATR), right cingulum, right inferior longitudinal fasciculus and right corticospinal tract (CT) 2. follow-up: ↑FA: in the ATR increased more in patients than in controls 3. The amisulpride dose correlated positively with FA changes in the right CT | 9 |
| Wang, et al. (2013)(20) | 35 (16) FES, 23.84±6.96 22 (14) HC, 22.41±5.96 | 7.26 ±5.32 | naïve | DSM-IV schizophrenia or schizophreniform psychosis | 6 weeks | FGA/SGA: Risperidone  Olanzapine  Quetiapine  Sulpiride  Aripiprazole  Haloperidol | 1. baseline: ↓FA: right posterior cingulate gyrus, the right anterior corona radiata and the precentral gyrus 2. follow-up (FES vs. HC): ↓FA: bilateral anterior cingulate gyrus and the right anterior corona radiata of the frontal lobe | 9 |
| Hu,et.al.(2016)(21) | 42 (27) FES, 24.86 ± 4.80 38 (25) HC, 24.76 ± 4.56 | 8.38±2.61 | naïve | DSM-IV-TR | 8 weeks | SGA: Risperidone | 1. baseline: deficient nodal topological properties primarily in prefrontal gyrus and limbic system components such as the bilateral anterior and posterior cingulate.  2. Treatment with risperidone normalized topological parameters in the limbic system 3. The enhancement of limbic system positively correlated with the reduction in PANSS-positive symptoms. | 9 |
| Hu,et.al.(2016)(22) | 42 (27) FES, 24.86 ± 4.80 38 (25) HC, 24.76 ± 4.56 | 8.38±2.61 | naïve | DSM-IV-TR | 8 weeks | SGA: Risperidone | 1. baseline: ↑fALFF: left caudate 2. follow-up: ↑fALFF: right caudate and bilateral putamen; ↑ReHo: right caudate and left putamen 3. Greater increase of fALFF in the left putamen correlated with less improvement in positive symptoms. | 9 |
| Wu, et.al. (2019)(23) | 32 (16) FES, 30.94 ± 8.25 32 (21) HC, 31.37 ± 7.84 | 8.91 ± 6.39 | naïve | DSM-IV | 1week/8 weeks | SGA: Olanzapine | 1. baseline: ↑fALFF: bilateral putamen 2. follow-up: 1 week: ↓fALFF: bilateral putamen (normalized) 3. The SVR analysis found a significantly positive relationship between the reduction in fALFF after 1 week of treatment and the improvement in positive symptoms after 8 weeks of treatment | 9 |
| Lui, et al. (2010)(24) | 30 (12) FES, 24.6 ±8.5 34 (13) HC, 25.0 ±8.0 | 7.8 ±12.4 | naïve | DSM-IV | 6-week | FGA/SGA:  Risperidone  Olanzapine  Clozapine  Quetiapine  Fumarate  Sulpiride  Aripiprazole. | 1. follow-up: ↑ALFF: bilateral prefrontal and parietal cortex, left superior temporal cortex, and right caudate nucleus; ↓FC: between 7 seeds. 2. Increased regional ALFF was associated with a reduction of clinical symptoms 3. Reductions in FC after treatment were correlated with the increases in ALFF values in all seed areas | 9 |
| Li, et al. (2016)(25) | 20 (6) FES, 22.9 ± 8.5 18 (7) HC, 22.4 ± 4.4 | 6.4 ±13.6 | naïve | DSM-IV | 1-year | FGA/SGA:  Risperidone  Quetiapine  Clozapine  Olanzapine  Sulpiride  Aripiprazole | 1. baseline: ↑ALFF: right occipital gyrus; ↓ALFF: right IPL and orbitofrontal cortex (OFC); ↓FC: bilateral IPLs 2. follow-up: ↑ALFF: right inferior parietal lobule (IPL) and orbitofrontal cortex (OFC); ↑FC: bilateral IPLs; ↓ALFF: right occipital gyrus returned to normal 3. The degree of alteration in ALFF values in the right OFC and occipital gyrus at baseline was significantly correlated with the magnitude of the normalization in those regions at 1-year follow-up. | 9 |
| Keedy, et al. (2015)(26) | 21 (16) FES, follow-up: 14 23.9 ± 7.9  21 (10) HC, follow-up: 12 24.7 ± 4.6 | NA | naïve/free | DSM-IV schizophrenia schizoaffective disorder depressed schizophreniform disorder | 4–6 weeks | SGA:  Risperidone  Aripiprazole | 1. Pretreatment, patients had reduced activation in the dorsal neocortical visual attention network. 2. Activation deficits were significantly reduced posttreatment. 3. Higher medication dose was associated with greater caudate activation at follow-up.  4. For the motor learning task, patients’ dorsolateral prefrontal cortex (DLPFC) was unimpaired prior to treatment but showed significantly reduced activation after treatment. | 8 |
| van Veelen, et al. (2011)(27) | 23 FES, 25.3± 4.6 24 HC, 24.5± 4.7 | 4.9 ± 4.4 | naïve | DSM-IV schizophreniform disorder or schizophrenia | 10 weeks | SGA:  Olanzapine  Risperidone  Quetiapine  Ziprasidone | 1. Non-responders showed a reduced practice effect in the DLPFC that was present already at baseline, which did not change after treatment. 2. A reduced practice effect in the DLPFC at baseline was found to be predictive of poor treatment response at 10 weeks. | 9 |
| Nielsen, et al. (2012)(28) | 23 (16) FES, 26.0 ± 6.7 24 (20) HC, 25.7 ± 5.9 | NA | naïve | ICD-10 schizophrenia or schizoaffective psychoses | 6-week | SGA: Antipsychotic compound amisulpride | 1. An attenuation of brain activation during reward anticipation in the ventral striatum, bilaterally 2. An increase in the anticipation-related functional magnetic resonance imaging signal and were no longer statistically distinguishable from healthy controls. 3. A correlation between the improvement of positive symptoms and normalization of reward-related activation. | 8 |
| Wulff, et al. (2020)(29) | 22 (10) FES, 23.4 ±4.7 23 (12) HC, 23.5 ±4.9 | 17.05±20.5 | naïve | ICD-10 | 6 weeks | SGA: Amisulpride | 1.Decreased BOLD signal in the caudate nucleus during salience anticipation in baseline not in follow-up. 2. There was a correlation between the treatment effect on positive symptoms and the improvement in BOLD signal 3.In patients characterised as responders based on their improvement on positive symptoms, there was a significant improvement in the BOLD signal, which was correlated with the occupancy of the D2 receptors.. | 9 |
| Han,et.al.(2020)(30) | 41 (26) FES, 24.98 ± 4.79 32 (21) HC, 25.12 ± 4.58 | 8.29 ± 2.58 | naïve | DSM-IV-TR schizophrenia | 8-week | SGA: Risperidone | 1. baseline: ↓FC: between distinct striatum sub-regions and the salience network 2. Follow-up: ↑FC: right dorsal rostral putamen and right anterior insular cortex, right dorsal caudal putamen and right supplementary motor area, left ventral rostral putamen and left superior frontal gyrus 4. Dorsal striatal pathways at baseline predicted negative symptom reduction while ventral striatal pathways predicted the positive symptom reduction. 5. The resilience of altered FC correlated with corresponding symptom improvements after treatment. | 9 |
| Li,et.al.(2020)(31) | 32 (16) FES,  30.94 ± 8.25 32 (21) HC, 31.37 ± 7.84 | 8.91 ± 6.39 | naïve | DSM-IV schizophrenia | 1 week/8 weeks | SGA: Olanzapine | 1. baseline: ↑GFC: bilateral anterior cingulate cortex (ACC)  2. follow-up: 1 week: ↓GFC: bilateral anterior cingulate cortex (ACC) 3. The SVR analysis suggested a positive relationship between GFC changes in bilateral ACC at week 1 and improvement in negative symptoms at week 8 | 9 |
| Wang,et.al.(2017)(32) | 33 (15) FES, 28.9 ± 5.7 33 (15) HC, 27.5 ± 4.9 | 8.4 ± 7.2 | naïve/free | DSM-IV-TR schizophrenia, schizophreniform disorder | 6–8 weeks | SGA:  Risperidone  Paliperidone  Olanzapine  Quetiapine  Aripiprazole  Ziprasidone | 1. In the DMN, the patients exhibited increased FC after treatment in the right superior temporal gyrus, right medial frontal gyrus, and left superior frontal gyrus and decreased FC in the right posterior cingulate/precuneus;In the SN, the patients exhibited decreased FC in the right cerebellum anterior lobe and left insula 2. The FC in the right posterior cingulate/precuneus (PCC/PCUN) in the DMN negatively correlated with the difference between the Clinical Global Impression (CGI) score pre/post-treatment and negative trends with the difference in the Positive and Negative Syndrome Scale (PANSS) total score pre/post-treatment and the difference in PANSS-positive symptom scores 3. These findings suggest that atypical antipsychotics could regulate the FC of certain key brain regions within the DMN in early-phase schizophrenia, which might be related to symptom improvement. | 8 |
| Duan, et.al. (2020)(33) | 42 (21) FES, 25.03 ± 4.71 33 (20) HC, 24.91 ± 4.48 | 8.03 ± 2.54 | naïve | DSM-IV | 8-week | SGA: Risperidone | 1. baseline: ↓FC: three PMC seeds with several brain regions (target regions) 2. follow-up: ↑FC: between the PCC and the bilateral thalamus and the left lingual gyrus (LG), the PCC and precuneus showed no changes. 3. A positive correlation between change of PANSS-total score and change of PCC-LG connectivity | 9 |
| Duan, et.al. (2020)(34) | 42 (27) FES, 24.86 ± 4.80 38 (25) HC, 24.76 ± 4.56 | 8.38 ± 2.61 | naïve | DSM-IV | 8-week | SGA: Risperidone | 1. baseline: ↓dFC: between the insular subdivisions and the precuneus, supplementary motor area and temporal cortex; ↑dFC: between the insular subdivisions and parietal cortex 2. follow-up: Abnormal connections were normalized 3. The normalized connections were accompanied by a significant improvement in positive symptoms | 9 |
| Zong,et.al.(2019)(35) | 42 (27) FES, 24.86 ± 4.80  38 (25) HC, 24.76 ± 4.56 | 8.38 ± 2.61 | naïve | DSM-IV-TR schizophrenia | 8 weeks | SGA: Risperidone | 1. baseline: ↓FC: posterior cingulate cortex/precuneus (PCC/PCUN) and medial prefrontal cortex (mPFC) 2. follow-up: ↑FC: PCC/PCUN 3. Increases in FC between PCC/PCUN and mPFC correlated with improvement in positive symptoms. 4. Anatomical and functional connectivity after treatment within the default mode network (DMN) were dissociated by modality | 9 |
| zhang,et.al.(2019)(36) | 60 (24) FES, 25.60 ± 6.99  60 (29) HC, 25.03 ± 6.41 | 8.55 ± 9.47 6.37 ± 3.74 | naïve | DSM-IV | 2-month | Antipsychotic (NA) | 1. Significantly disrupted functional connectivity in the sensory-motor network. 2. The degree of impairment reflected the duration of untreated psychosis and motor-related symptoms. 3. It further predicted the improvement of positive scores after medication. | 8 |

Abbreviations: FES: first episode schizophrenia. HC: healthy control. NA: not available. DSM: Diagnostic and Statistical Manual of Mental Disorders. ICD-10: International Classification of Diseases. PANSS: Positive and Negative Syndrome Scale. GMV: grey matter volume. WMV: white matter volume. FA: fractional anisotropy. ALFF: amplitude of low frequency fluctuations. ReHo: regional homogeneity. FC: functional connectivity. GFC: global functional connectivity. FGA: first-generation antipsychotics. SGA: second-generation antipsychotics. BOLD: Blood Oxygen Level-Dependent. NOS:Newcastle-Ottawa Scale.

↓decreased. ↑ increased.

**References:**

1. Li W, Li K, Guan P, Chen Y, Xiao Y, Lui S, et al. Volume alteration of hippocampal subfields in first-episode antipsychotic-naive schizophrenia patients before and after acute antipsychotic treatment. Neuroimage Clin. 2018;20:169-176.

2. Rizos E, Papathanasiou MA, Michalopoulou PG, Laskos E, Mazioti A, Kastania A, et al. A longitudinal study of alterations of hippocampal volumes and serum BDNF levels in association to atypical antipsychotics in a sample of first-episode patients with schizophrenia. Plos One. 2014;9:e87997.

3. Ebdrup BH, Skimminge A, Rasmussen H, Aggernaes B, Oranje B, Lublin H, et al. Progressive striatal and hippocampal volume loss in initially antipsychotic-naive, first-episode schizophrenia patients treated with quetiapine: relationship to dose and symptoms. The international journal of neuropsychopharmacology. 2011;14:69-82.

4. Lei W, Kirkpatrick B, Wang Q, Deng W, Li M, Guo W, et al. Progressive brain structural changes after the first year of treatment in first-episode treatment-naive patients with deficit or nondeficit schizophrenia. Psychiatry research Neuroimaging. 2019;288:12-20.

5. Poeppl TB, Frank E, Schecklmann M, Kreuzer PM, Prasser SJ, Rupprecht R, et al. Amygdalohippocampal neuroplastic changes following neuroleptic treatment with quetiapine in first-episode schizophrenia. The international journal of neuropsychopharmacology. 2014;17:833-843.

6. Andersen HG, Raghava JM, Svarer C, Wulff S, Johansen LB, Antonsen PK, et al. Striatal Volume Increase After Six Weeks of Selective Dopamine D(2/3) Receptor Blockade in First-Episode, Antipsychotic-Naïve Schizophrenia Patients. Front Neurosci. 2020;14:484.

7. Yue Y, Kong L, Wang J, Li C, Tan L, Su H, et al. Regional Abnormality of Grey Matter in Schizophrenia: Effect from the Illness or Treatment? Plos One. 2016;11:e0147204.

8. Molina V, Reig S, Sanz J, Palomo T, Benito C, Sanchez J, et al. Increase in gray matter and decrease in white matter volumes in the cortex during treatment with atypical neuroleptics in schizophrenia. Schizophrenia Research. 2005;80:61-71.

9. Deng MY, McAlonan GM, Cheung C, Chiu CPY, Law CW, Cheung V, et al. A naturalistic study of grey matter volume increase after early treatment in anti-psychotic na < ve, newly diagnosed schizophrenia. Psychopharmacology. 2009;206:437-446.

10. Heitmiller DR, Nopoulos PC, Andreasen NC. Changes in caudate volume after exposure to atypical neuroleptics in patients with schizophrenia may be sex-dependent. Schizophrenia Research. 2004;66:137-142.

11. Massana G, Salgado-Pineda P, Junque C, Perez M, Baeza I, Pons A, et al. Volume changes in gray matter in first-episode neuroleptic-naive schizophrenic patients treated with risperidone. Journal of clinical psychopharmacology. 2005;25:111-117.

12. Li M, Chen Z, Deng W, He Z, Wang Q, Jiang L, et al. Volume increases in putamen associated with positive symptom reduction in previously drug-naive schizophrenia after 6 weeks antipsychotic treatment. Psychol Med. 2012;42:1475-1483.

13. Li M, Li X, Das TK, Deng W, Li Y, Zhao L, et al. Prognostic Utility of Multivariate Morphometry in Schizophrenia. Front Psychiatry. 2019;10:245.

14. McCormick L, Decker L, Nopoulos P, Ho BC, Andreasen N. Effects of atypical and typical neuroleptics on anterior cingulate volume in schizophrenia. Schizophrenia Research. 2005;80:73-84.

15. Théberge J, Williamson KE, Aoyama N, Drost DJ, Manchanda R, Malla AK, et al. Longitudinal grey-matter and glutamatergic losses in first-episode schizophrenia. The British journal of psychiatry : the journal of mental science. 2007;191:325-334.

16. Mane A, Falcon C, Mateos JJ, Fernandez-Egea E, Horga G, Lomena F, et al. Progressive gray matter changes in first episode schizophrenia: a 4-year longitudinal magnetic resonance study using VBM. Schizophr Res. 2009;114:136-143.

17. Girgis RR, Diwadkar VA, Nutche JJ, Sweeney JA, Keshavan MS, Hardan AY. Risperidone in first-episode psychosis: A longitudinal, exploratory voxel-based morphometric study. Schizophrenia Research. 2006;82:89-94.

18. Meng L, Li K, Li W, Xiao Y, Lui S, Sweeney JA, et al. Widespread white-matter microstructure integrity reduction in first-episode schizophrenia patients after acute antipsychotic treatment. Schizophr Res. 2019;204:238-244.

19. Ebdrup BH, Raghava JM, Nielsen MO, Rostrup E, Glenthoj B. Frontal fasciculi and psychotic symptoms in antipsychotic-naive patients with schizophrenia before and after 6 weeks of selective dopamine D2/3 receptor blockade. J Psychiatry Neurosci. 2016;41:133-141.

20. Wang Q, Cheung C, Deng W, Li M, Huang C, Ma X, et al. White-matter microstructure in previously drug-naive patients with schizophrenia after 6 weeks of treatment. Psychol Med. 2013;43:2301-2309.

21. Hu M, Zong X, Zheng J, Mann JJ, Li Z, Pantazatos SP, et al. Risperidone-induced topological alterations of anatomical brain network in first-episode drug-naive schizophrenia patients: a longitudinal diffusion tensor imaging study. Psychol Med. 2016;46:2549-2560.

22. Hu ML, Zong XF, Zheng JJ, Pantazatos SP, Miller JM, Li ZC, et al. Short-term Effects of Risperidone Monotherapy on Spontaneous Brain Activity in First-episode Treatment-naive Schizophrenia Patients: A Longitudinal fMRI Study. Scientific reports. 2016;6:34287.

23. Wu R, Ou Y, Liu F, Chen J, Li H, Zhao J, et al. Reduced Brain Activity in the Right Putamen as an Early Predictor for Treatment Response in Drug-Naive, First-Episode Schizophrenia. Front Psychiatry. 2019;10:741.

24. Lui S, Li T, Deng W, Jiang L, Wu Q, Tang H, et al. Short-term effects of antipsychotic treatment on cerebral function in drug-naive first-episode schizophrenia revealed by "resting state" functional magnetic resonance imaging. Arch Gen Psychiatry. 2010;67:783-792.

25. Li F, Lui S, Yao L, Hu J, Lv P, Huang X, et al. Longitudinal Changes in Resting-State Cerebral Activity in Patients with First-Episode Schizophrenia: A 1-Year Follow-up Functional MR Imaging Study. Radiology. 2016;279:867-875.

26. Keedy SK, Reilly JL, Bishop JR, Weiden PJ, Sweeney JA. Impact of antipsychotic treatment on attention and motor learning systems in first-episode schizophrenia. Schizophr Bull. 2015;41:355-365.

27. van Veelen NM, Vink M, Ramsey NF, van Buuren M, Hoogendam JM, Kahn RS. Prefrontal lobe dysfunction predicts treatment response in medication-naive first-episode schizophrenia. Schizophr Res. 2011;129:156-162.

28. Nielsen MO, Rostrup E, Wulff S, Bak N, Broberg BV, Lublin H, et al. Improvement of brain reward abnormalities by antipsychotic monotherapy in schizophrenia. Arch Gen Psychiatry. 2012;69:1195-1204.

29. Wulff S, Nielsen M, Rostrup E, Svarer C, Jensen LT, Pinborg L, et al. The relation between dopamine D(2) receptor blockade and the brain reward system: a longitudinal study of first-episode schizophrenia patients. Psychol Med. 2020;50:220-228.

30. Han S, Becker B, Duan X, Cui Q, Xin F, Zong X, et al. Distinct striatum pathways connected to salience network predict symptoms improvement and resilient functioning in schizophrenia following risperidone monotherapy. Schizophr Res. 2020;215:89-96.

31. Li H, Ou Y, Liu F, Chen J, Zhao J, Guo W, et al. Reduced connectivity in anterior cingulate cortex as an early predictor for treatment response in drug-naive, first-episode schizophrenia: A global-brain functional connectivity analysis. Schizophr Res. 2020;215:337-343.

32. Wang Y, Tang W, Fan X, Zhang J, Geng D, Jiang K, et al. Resting-state functional connectivity changes within the default mode network and the salience network after antipsychotic treatment in early-phase schizophrenia. Neuropsychiatric disease and treatment. 2017;13:397-406.

33. Duan X, Hu M, Huang X, Dong X, Zong X, He C, et al. Effects of risperidone monotherapy on the default-mode network in antipsychotic-naive first-episode schizophrenia: Posteromedial cortex heterogeneity and relationship with the symptom improvements. Schizophr Res. 2020.

34. Duan X, Hu M, Huang X, Su C, Zong X, Dong X, et al. Effect of Risperidone Monotherapy on Dynamic Functional Connectivity of Insular Subdivisions in Treatment-Naive, First-Episode Schizophrenia. Schizophr Bull. 2020;46:650-660.

35. Zong X, Hu M, Pantazatos SP, Mann JJ, Wang G, Liao Y, et al. A Dissociation in Effects of Risperidone Monotherapy on Functional and Anatomical Connectivity Within the Default Mode Network. Schizophr Bull. 2019;45:1309-1318.

36. Zhang Y, Xu L, Hu Y, Wu J, Li C, Wang J, et al. Functional Connectivity Between Sensory-Motor Subnetworks Reflects the Duration of Untreated Psychosis and Predicts Treatment Outcome of First-Episode Drug-Naive Schizophrenia. Biol Psychiatry Cogn Neurosci Neuroimaging. 2019;4:697-705.
